# Comparative genomics of *Salmonella enterica* serovars Derby and Mbandaka, two prevalent serovars associated with different livestock species in the UK

**DOI:** 10.1186/1471-2164-14-365

**Published:** 2013-05-31

**Authors:** Matthew R Hayward, Vincent AA Jansen, Martin J Woodward

**Affiliations:** 1 Animal Health and Veterinary Laboratories Agency, Woodham Lane, New Haw, Addlestone, Surrey KT15 3NB, UK; 2 School of Biological Sciences, Royal Holloway University of London, Egham, Surrey, TW20 0EX, UK; 3 Department of Food and Nutritional Sciences, Reading University, Whiteknights, Reading, RG6 6AP, UK

**Keywords:** *Salmonella*, *S*. Derby, *S*. Mbandaka, Functional genomics, SPI-23, Host adaptation

## Abstract

**Background:**

Despite the frequent isolation of *Salmonella enterica* sub. *enterica* serovars Derby and Mbandaka from livestock in the UK and USA little is known about the biological processes maintaining their prevalence. Statistics for *Salmonella* isolations from livestock production in the UK show that *S*. Derby is most commonly associated with pigs and turkeys and *S*. Mbandaka with cattle and chickens. Here we compare the first sequenced genomes of *S*. Derby and *S*. Mbandaka as a basis for further analysis of the potential host adaptations that contribute to their distinct host species distributions.

**Results:**

Comparative functional genomics using the RAST annotation system showed that predominantly mechanisms that relate to metabolite utilisation, *in vivo* and *ex vivo* persistence and pathogenesis distinguish *S*. Derby from *S*. Mbandaka. Alignment of the genome nucleotide sequences of *S*. Derby D1 and D2 and *S*. Mbandaka M1 and M2 with S*almonella* pathogenicity islands (SPI) identified unique complements of genes associated with host adaptation. We also describe a new genomic island with a putative role in pathogenesis, SPI-23. SPI-23 is present in several *S. enterica* serovars, including *S*. Agona, *S*. Dublin and *S*. Gallinarum, it is absent in its entirety from *S*. Mbandaka.

**Conclusions:**

We discovered a new 37 Kb genomic island, SPI-23, in the chromosome sequence of *S.* Derby, encoding 42 ORFS, ten of which are putative TTSS effector proteins. We infer from full-genome synonymous SNP analysis that these two serovars diverged, between 182kya and 625kya coinciding with the divergence of domestic pigs. The differences between the genomes of these serovars suggest they have been exposed to different stresses including, phage, transposons and prolonged externalisation. The two serovars possess distinct complements of metabolic genes; many of which cluster into pathways for catabolism of carbon sources.

## Background

*Salmonella enterica* subspecies *enterica* is an important zoonotic pathogen of warm-blooded vertebrates, with both a broad host species range and geographical distribution. The subspecies is divided into over 1530 serovars based on the different epitopes of two surface antigens, the O lipopolysaccharide, and H flagellum of which there are commonly two phases [1].

Some serovars display association with a particular set of hosts that may be stable over many decades and large geographical distances suggesting a level of adaptation or restriction [2]. With regard to serovars *S.* Derby and *S.* Mbandaka, both serovars are isolated with similar frequency in the UK and USA. Annually compiled statistics from several sources [3,4] (HPA personal communication) showed that, whilst both serovars can readily cause disease in people, incidences in livestock show differing host associations. In the UK, for example, approximately 50% and 40% of incidences of *S*. Derby are in turkeys and pigs, respectively, and approximately 20% and 65% of incidences of *S.* Mbandaka are from cattle and chickens, respectively [3]. In the USA approximately 80% of isolation of *S*. Derby are from pigs, while only 3% of isolations were from turkeys, 27% and 25% of *S*. Mbandaka isolations are from cattle and chickens. Unlike in the UK in the USA *S.* Mbandaka is isolated from pigs comprising 14% of the total [4]. These host distributions have been maintained for over a decade and on two continents which gives rise to at least two hypotheses. First, is it possible that the differences in host association may relate to production systems and that these serotypes posses similar functional capabilities. Second, is it possible that the differences in host association reflect functional differences between serovars or genovars therein, whereby there exist bacterially encoded mechanisms that maintain these patterns. As a starting point to tackle these opposing hypotheses, we present the first full chromosome sequence of two UK isolates of both *S*. Derby and *S*. Mbandaka. We use functional genomics to describe genome features and to identify genes that are unique with a view to gaining insights into potential genetic components that contribute to the species distributions described above.

## Results and discussion

The chromosomes of two strains of *S*. Derby and *S*. Mbandaka were sequenced and compared with the goal of identifying potential mechanistic differences between the two serovars that could explain their skewed isolation frequencies from subsets of livestock species in the UK. Strains were obtained from background monitoring performed by the Animal Health and Veterinary Laboratories Agency (AHVLA) in the UK between 2000 and 2010. In total 28 strains were selected spanning the decade and from differing geographic points of isolation across the UK (locations not shown due to sensitivity of data). The hosts of isolation of the selected strains were chosen to reflect the two most common hosts of each serovar, for *S*. Derby these were pigs and turkeys and for *S*. Mbandaka cows and chickens. Two isolates of each serovar isolated from separate geographical locations, with the same host species, and identical MLST sequence types (*S*. Derby strains ST40 and *S*. Mbandaka strains ST900) were chosen for full genome sequencing. We recognised that in the absence of information regarding the pan-genome of the population, that by comparing just two isolates of each serovar, we could potentially infer, incorrectly, that differences in gene complement between isolates of the same serovar isolated from different hosts were adaptations to these different hosts. The selection was therefore made with the aim of better understanding the genomic differences between strains which would typically be considered clonal. *S*. Derby strains D1 and D2 were both isolated in 2008 from porcine hosts. *S*. Mbandaka M1 and M2 were isolated from cattle in 2008 and 2009 respectively. No research has previously been performed on these strains.

### General genome features of *S.* Derby D1 and D2 and *S.* Mbandaka M1 and M2

**Figure 1:**
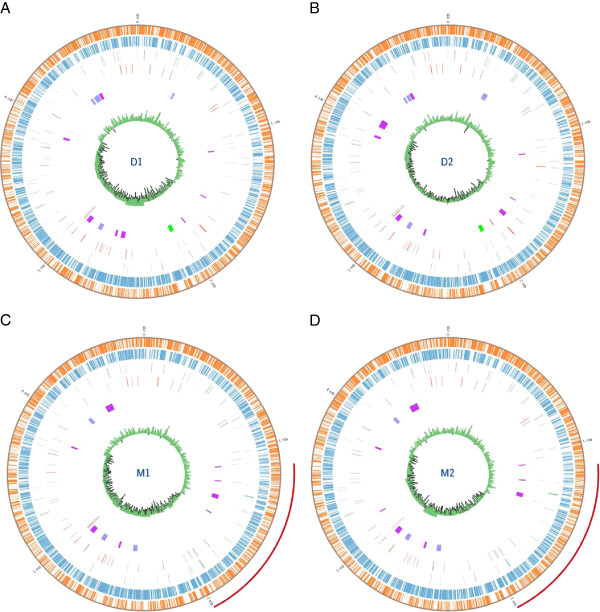
**Chromosome annotation maps of newly sequenced isolates. A**) *S*. Derby D1, **B**) *S*. Derby D2, **C**) *S*. Mbandaka M1 and **D**) *S*. Mbandaka M2. The two outer tracks represent ORFs identified by RAST in forward and reverse DNA strands. The third and fourth tracks display non-coding RNAs in forward and reverse confirmation. The fifth track shows, SPI-23 (green) in maps A and B, prophage (mauve), *Salmonella *pathogenicity islands (purple) and CRISPR operons (burgundy). The sixth track shows the GC base composition under a 1000bp moving window. The additional red track outside of maps C and D indicates the location of a unique 860 kbp sequence inversion.

*S.* Derby strains D1 and D2 possessed chromosomes of 4.86 Mb nucleotides in length with a GC skew of 51.16% and 51.46% respectively. The RAST annotation system predicted that the chromosome sequence of *S*. Derby D1 encodes 4720 genes and the sequence of D2 4717 genes. The chromosome of *S.* Mbandaka strains M1 and M2 were both 4.72 MB nucleotides in length with a GC skew of 51.91% and 52.01% respectively. These were predicted to encode 4616 and 4619 genes respectively. Interestingly all four chromosomes contain different numbers of RNA coding sequences, D1 contains 69, D2 contains 73, M1 contains 74 and M2 contains 75 (Figure 1). RNA sequences are frequently sights for integration of horizontally acquired DNA sequences, in some cases leading to duplication of the RNA [5-7]. The difference in the number of RNAs in each genome could reflect a difference in evolutionary potential of each chromosome.

### *S*. Mbandaka contains a large sequence inversion

*S.* Mbandaka contains a 860Kb sequence inversion between a mobile element protein and tRNA-ser-GGA (located between base 1086415 and 1947250 of M1, and 1132370 and 1992477 of M2) which was also found in *S*. Choleraesuis SC-B67, and was absent from *S.* Derby (Figure 1) and other sequenced *S. enterica* serovars including *S.* Agona SL483, *S*. Dublin CT02021853, *S.* Enteritidis P125109, *S.* Gallinarum 28791 and *S.* Typhimurium LT2 and SL1344. This region codes for 909 genes identified by the RAST gene caller. Large sequence inversions have a significant impact on the transcript composition of the cell during replication, as those genes closer to the origin of replication are present in duplicate for a longer period of time than those genes closer to the terminus of replication [8,9]. The effects of increased gene dosage during replication are most noticeable when bacteria are growing at an optimal rate [10]. In *Escherichia coli* DNA replication from the origin of replication to terminus of replication takes 22 minutes during a 40 minute cell cycle when grown in LB broth at 37°C [11]. If we apply this duration to the inversion found in *S*. Mbandaka M1 and M2, where almost a quarter of the chromosome is in a different orientation to *S*. Derby D1 and D2, then there is an 8.6 minute difference between gene duplication events of the genes adjacent to the sites of inversion. These genes are therefore in duplicate and the other genes in singlet for 21% of the cell cycle. In *S*. Derby the ten genes closest to the mobile genetic element signifying the start of the inverted sequences do not pertain to a common mechanism. Though interestingly, amongst these ten genes is a permease of the drug/metabolite transporter (DMT) superfamily which in *S.* Mbandaka occupies the very furthest gene in the inversion. The ten genes at the terminus of the inversion in the chromosome of *S*. Derby D1 and D2 comprise of two operons, the Csg-curli operon (four genes) and Ycd-swarming operon (five genes). The most interesting aspect of these two operons is that they are associated with two diametrically opposed mechanisms; the curli operon is associated with biofilm development in a sessile population and the swarming operon is associated with directed movement of the bacterial population, both using quorum sensing [12,13]. Both are population scale emergent phenotypes of gene regulation at a single cell level. *csgD* found in the curli operon regulates both curli expression and cellulose secretion, the main components of biofilms in *Salmonella enterica*[14]. The ten genes at the centre of inversion and therefore in similar dosage throughout replication in *S*. Derby and *S*. Mbandaka are genes for formate dehydrogenase alpha, beta and gamma subunits which form a single transmembrane enzyme [15]. Also contained within this region is a permease of the drug/metabolite transporter (DMT) superfamily. The ten genes at either end and in the centre of the sequence inversion can be found listed in the supplementary materials [Additional file 1].

### Summary of functional annotation

The chromosome of *S.* Derby is 140 kb longer than that of *S.* Mbandaka, coding for 100 additional genes. RAST annotation was performed on 9/10/12 and achieved 67% coverage with FIGfam subsystems of the *S*. Derby D1 and D2 chromosomes and 68% of the *S*. Mbandaka M1 and M2 chromosomes [16]. FIGfam clusters genes based on protein sequence similarity. From this the function of a novel gene may be inferred. These genes are then clustered into hierarchical subsystems that display increasing functional breadth [17]. As the database has developed the subsystem coverage of the four genomes presented here has markedly increased by 8-9% over the same annotation performed on the 9/10/11. The most recent annotation is available through the following RAST IDs: D1 [RAST: 28144.16], D2 [RAST: 28144.17], M1 [RAST: 192954.16] and M2 [RAST: 192954.17] so that the chromosome can be re-annotated as the RAST databases are updated. The number of hypothetical genes between the annotations remained constant for each chromosome. In all cases almost a quarter of the genome annotation was found to be of hypothetical gene status. *S*. Derby contains 96 hypothetical/putative proteins which share less than 90% amino acid sequence homology with open reading frames in *S.* Mbandaka. *S.* Mbandaka contains 155 unique hypothetical/putative proteins.

### Intra-serovar differences in the complement of functionally unique genes

The majority of the diversity between strains of the same serovars was in the complement of phage associated genes. All intra-serovar differences in gene complement can be found listed in the supplementary materials [Additional file 1]. Between the *S*. Derby isolates, D1 contains a single unique gene for an aconitate hydratase 2 (EC 4.2.1.3) associated with glyoxylate bypass. D2 contains 11 unique genes, of which five are associated with phage, the remaining are associated with metabolism. A single gene which is associated with the ribosome at stationary growth phase is absent from D1. There is less diversity between isolates of *S*. Mbandaka. M1 contains a single additional gene which encodes a phage tail fibre protein. M2 contains six additional genes, two for cytochrome-c biosynthesis, two phage genes and a gene encoding a 2,5-diketo-D-gluconic acid reductase B (EC 1.1.1.274).

### Inter-serovar differences of functionally unique genes

Genes pertaining to metabolite utilisation, prophage, CRISPR spacers and *Salmonella* pathogenicity islands will be dealt with separately. The following summarises the genes that do not fit into these categories. All inter-serovar differences in gene complement can be found listed in the supplementary materials [Additional file 1].

### *Salmonella* Derby

*S*. Derby contains 16 genes that are functionally unique. Of these 16 genes 12 are distributed between two operons. One is the *mer* operon conveying mercury resistance. This consists of five genes including *merC* and *merT* transport proteins, which actively take up toxic mercuric cations (Hg^+2^) for subsequent reduction to non-toxic metallic mercury (Hg^0^) [18]. Mecuric cations enter the food chain from several sources, including fish, poultry and meat. Animals feed is frequently supplemented in the UK with fish meal which is high in mercury. Fish meal contains the second highest concentration of mercury per kg in animal feed/ pet food in Europe, with fish oil containing the highest concentration [19]. The second operon, is a CRISPR operon made up of seven genes, one of putative status and *cse1-cse4*, *cas1*, *cas2* and *cas5e*. Of the remaining four genes one is the gene for an UDP-galactopyranose mutase (EC 5.4.99.9) which is associated with the biosynthesis of alpha-D-galactofuranose, a component of the O-antigen in *Salmonella enterica* groups B, C2, D and E [20].

### *Salmonella* Mbandaka

*S.* Mbandaka contains a cluster of four type VII secretion system Yad fimbrial chaperone proteins. Two genes with the same function from the HtrE fimbrial cluster sit approximately 100 KB away. A further 2 MB away at 4.5U sits a cluster of three beta-fimbriae genes also associated with type VII secretion. Three sialic acid metabolism genes associated with capsule production, *nanC*, *nanM* and a hypothetical gene, are clustered around 1U. Two cell death toxin-antitoxin genes, *phd* and *doc,* are unique to M1 and M2, and may be involved in plasmid addiction systems. Two genes associated with reduction in mutation rate due to exposure to bile salts are absent from *S.* Derby. These genes, *umuC* and *umuD* are part of the SOS DNA repair response and form DNA polymerase V. It has been shown in *E. coli* that in the absence of *umuC* genomic lesions are not repaired correctly by DNA polymerase III and can leave frame shift mutations which lead to pseudogene formation. DNA polymerase V has a higher rate of single nucleotide mutations than DNA polymerase III [21,22]. This could lead to a higher rate of pseudogene formation in *S*. Mbandaka strains and SNP formation in *S*. Derby strains. However, this would need to be confirmed through further analyses.

There are only seventeen genes that are unique in function to either *S.* Derby or *S.* Mbandaka that are not clustered. Of these seventeen genes *S.* Mbandaka contains seven unique genes related to biogenesis of cytochrome-c, specifically the maturation of the molecule, and are spread across the chromosome. The genes *ccmB*, *ccmC* and *ccmD* convey the heme-b group to the product of CcmE, a monotopic membrane protein [23]. The products of *ccmF*, *ccmG* and *ccmH* complex with CcmE to convey the heme-b group to the apocytochrome-c precursor of cytochrome-C [24,25]. Though these genes are ubiquitous amongst Gram negative bacteria, strains of *E. coli* have been discovered that lack the *ccm* operon and yet are able to synthesis cytochrome-c containing heme-b [26].

### Differences in metabolic gene complement between *S*. Derby and *S*. Mbandaka

Fourteen genes were identified by RAST subsystem annotations as being involved in primary or secondary metabolism which were found to differ between *S.* Derby and *S.* Mbandaka. Six of these genes belong to *S.* Mbandaka are associated with D-galactonate catabolism, this includes uptake, regulation and processing into central carbon metabolism. *S.* Derby contains six genes for the uptake and catabolism of six different carbon sources, this comprises an asparagine synthetase (EC 6.3.5.4), a hydroxyaromatic non-oxidative decarboxylase protein D (EC 4.1.1.-), a protein fumarylacetoacetate of the hydrolase family, phosphatase NagD predicted to act inN-acetylglucosamine utilization subsystem, an aconitate hydratase 2 (EC 4.2.1.3), a galactose-specific IIA component (EC 2.7.1.69) and the large subunit of a glycerol dehydratase reactivation factor.

### Metabolic pathways

The biological significance of the differences in metabolic genes was elaborated through construction of metabolic models from the genome sequences using SEEDmodel [16]. These differences were then elaborated in context of the surrounding reactions. Metabolic reconstructions curated with phenotypic data are underway to better understand the effect of secondary metabolism on the optimal growth rate of *S*. Derby D1 and *S*. Mbandaka M1.

### Alanine, aspartate and glutamate metabolism map 00250 created 1/6/12

*S*. Derby lacks a single gene, an aspartate—ammonia ligase (EC6.3.1.1) for the conversion of L-aspartate to L-asparagine. The same reaction is achievable through two additional reactions utilising an asparaginase/glutaminase (EC3.5.1.38) and an L-asparaginase (EC3.5.1.1) which are also present in *S*. Mbandaka.

### Galactose metabolism map 00052 created 31/5/12

The three genes encoding products needed to feed D-galactonate into glycolysis (EC 4.2.1.6, EC 2.7.1.58 and EC 4.1.21) by conversion to D-glyceraldehyde-3P are present on the chromosome of *S.* Mbandaka and absent from that of *S.* Derby. There are no alternative routes from D-galactonate to glycolysis.

### Nitrogen metabolism map 00910 created 21/8/12

A gene coding for the enzyme L-glutamine amido-ligase that converts L-glutamine to L-glutamate using one molecule of H_2_O in the process (EC 6.3.5.4) is missing from the chromosome of *S.* Derby D1. All strains contain a gene that catalyses the same reaction but with the requirement of a molecule of NADP^+^ as opposed to one of H_2_O (EC 1.4.1.13).

### Starch and sucrose metabolism map 00500 created 9/7/12

A single reaction is missing from *S.* Mbandaka in this map for the conversion of alpha-D-Glucose-1-P to CDP-glucose (EC 2.7.7.33); there is no route to this compound other than this on the map. The CDP-glucose then leads into amino sugar and nucleotide sugar metabolism map 00520 created 19/1/10. In this map there is an additional reaction from CDP-glucose leading to CDP-4-keto-6-deoxy-D-Glucose missing in *S*. Mbandaka. This reaction is catalysed by the enzyme RfbG, a CDP-glucose 4,6-dehydratase (EC 4.2.1.45) which is found in *Salmonella enterica* groups A, B, C2, C3, D1 and D2 and required for binding of the O antigen to the core oligosaccharide [27,28]. *S*. Mbandaka is a member of *S. enterica* group C1.

### Streptomycin biosynthesis pathway map 00521 created 27/12/10

Two steps from D-glucose-1-P are present in both serovars (EC 2.7.7.24 and EC 4.2.1.46), following on from the terminal product of this reaction, two additional steps that lead to dTDP-L-rhamnose are missing in *S.* Mbandaka (EC 5.1.3.13 and EC 1.1.1.133). dDTP-L-Rhamnose feeds directly into novobiocin biosynthesis, diverted out of the streptomycin biosynthesis pathway. *S*. Mbandaka is left with a product which feeds into polyketide sugar unit biosynthesis (Pathway 00523, created 14/3/12, polyketide sugar unit biosynthesis).

### *Salmonella* pathogenicity islands

The chromosome of *Salmonella enterica* comprises largely of a core sequence punctuated with horizontally acquired sequences [29]. The complement of genomic islands within the chromosome of *Salmonella enterica* can vary amongst isolates of the same serovar [30,31]. It has been postulated that the acquisition of horizontally acquired genes into a *Salmonella* pathogenicity island (SPI) led to the divergence of *Salmonella* from *Escherichia coli*[32,33]. *Salmonella* pathogenicity island 1 (SPI-1) is found in all serovars of *S. enterica* (with the exception of *S.* Seftenberg and *S*. Litchfield) and is highly conserved [32,34,35]. There are currently 22 published *Salmonella* pathogenicity islands identified from the genomes of *Salmonella enterica* and *Salmonella bongori*[36]. The gene content of some of these islands is highly plastic, as exemplified by the different gene complement of SPI-3 found in *S.* Dublin CT02021853 and *S.* Typhimurium LT2 [37]. The *Salmonella* pathogenicity islands are well characterised in terms of genetic composition and putative function but less so, with notable exceptions, for their role in pathogenicity [38,39]. Hence differences in SPI complement and gene content of D1, D2, M1 and M2 chromosomes may hint at mechanisms that maintain their respective host species range.

### Complete or absent *Salmonella* pathogenicity islands

SPIs 2 and 4 found in the genome of *S.* Choloreaesuis SC-B67 and SPI-18 from *S.* Typhi CT18 are complete in the genomes of *S.* Derby D1 and D2, and *S.* Mbandaka M1 and M2. SPI-7, 8, 10, 15, 16, 17, 19, 20, 21 and 22 were absent from both *S.* Derby D1 and D2, and *S.* Mbandaka M1 and M2 genomes.

### Variation in SPI-1of *S.* Derby and *S.* Mbandaka

SPI-1 in *S.* Mbandaka M1 and M2 shares 100% nucleotide sequence identity with *S.* Typhumirum LT2 with the addition of two ORFs coding for hypothetical proteins found in the SPI-1 of *S.* Choleraesuis SC-B67, SC2837 and SC2838 which are absent in *S.* Derby D1 and D2. *S.* Derby D1 and D2 lack three genes from SPI-1 of *S.* Typhimurium LT2, STM2901, STM2902 and STM2903 (Table 1). SIEVE an online server for the prediction of TTSS effector proteins, found that the *S.* Mbandaka M1 and M2 contained an ORF with 98% amino acid sequence homology with SC2837 from *S.* Choleraesuis SC-B67, is a likely candidate for an effector protein with a p-value of 0.003. With reference to well-characterised effector proteins, all four isolates contain intact versions of *sopB* and *sopE*. The two putative cytoplasmic proteins found in SPI-1 of *S.* Typhimurium LT2, STM2901 and STM2902 and here in *S.* Mbandaka M1 and M2 and not D1 and D2 are unlikely candidates for effector proteins with p-values of 0.142.

**Table 1:** Comparison of previously published genomic islands that distinguish between D1, D2, M1 and M2 in their gene complement

**SPI**	**Gene**	**D1**	**D2**	**M1**	**M2**	**Descriptor**	**Role in pathogenesis**
CS54	*ratA*	-	+	+	+	Ribosome association toxin	No role in virulence [10]
	*sivI*	-	+	+	+	Outer membrane protein	No role in virulence [10]
	*sivH*	-	+	+	+	Invasin-like	Colonising peyers patch (Mouse) [10]
SPI-1	STM2901	-	-	+	+	Putative cytoplasmic protein	NI [40]
	STM2902	-	-	+	+	Putative cytoplasmic protein	NI [40]
	STM2903	-	-	+	+	Putative cytoplasmic protein	NI [40]
	SC2837	-	-	+	+	Hypothetical protein	NI [41]
	SC2838	-	-	+	+	Hypothetical protein	NI [41]
SPI-3	Pseudo	+	+	-	-	NI	NI [42]
	*yadC*	+	+	-	-	Fimbrial like protein	Stress response [42,43]
	*yadK*	+	+	-	-	Fimbrial like protein	NI [42]
	*yadL*	+	+	-	-	Fimbrial like protein	NI [42]
	*yadN*	+	+	-	-	Fimbrial like protein	NI [42]
	*htrE*	+	+	-	-	Porin/ fimbrial assembly	High temperature resistance above 50°C [44]
	*ecpD1*	+	+	-	-	Pilin chaperone	Expressed with increasing temp above 22°C [44]
	*ecpD2*	+	+	-	-	Pilin chaperone	NI [42]
	Pseudo	+	+	-	-	NI	NI [42]
	*rhuM*	-	-	+	+	Cytoplasmic protein	Epithelial migration [45]
	STY4039	-	-	+	+	EnvR binding site	No role in virulence [46]
SPI-6	STY0296	+	+	-	-	Hypothetical protein	No role in virulence [46]
	STY0300 (*sirA*)	-	-	+	+	Transcription factor	Regulates expression of SPI1 and flagellum genes [47,48]
	STY0301 (*safC*)	-	-	+	+	Outer membrane usher protein	Up regulated during intracellular replication [49]
	STY0302 (*sciM*)	-	-	+	+	Hemolysin-coregulated protein	NI [50]
	STY0303 (*sciN*)	-	-	+	+	Outer membrane lipoprotein	Need for Type VI secretion, biofilm formation [51]
	STY0307	-	-	+	+	Hypothetical protein	NI [50]
	STY0311	-	-	+	+	Mannosyl-glycoprotein	NI [50]
	STY0312	-	-	+	+	Hypothetical protein	NI [50]
	STY0319	-	-	+	+	Rhs-family protein	NI [50]
	STY0320	-	-	+	+	Putative cytoplasmic protein	NI [50]
	STY0321	-	-	+	+	Rhs1 protein	NI [50]
	STY0322	-	-	+	+	Hypothetical protein	NI [50]
	STY0323	-	-	+	+	Hypothetical protein	NI [50]
	*safA*	-	-	+	+	Fimibrial usher protein	No effect in virulence in mice [50,52]
	Pseudo	-	-	+	+	NI	NI [46]
	*safB*	-	-	+	+	Periplasmic fimbrial chaperone protein	NI [46]
	*safC*	-	-	+	+	Outer membrane usher protein	Up regulated during intracellular replication [49]
	*safD*	-	-	+	+	Fimibrial usher protein	No effect in virulence in mice [52]
	STY0338	-	-	+	+	Periplasmic binding protein	NI [46,50]
	Pseudo	-	-	+	+	NI	NI [46]
	*sinR (pagN*)	-	-	+	+	HTH transcription factor	No effect on virulence in mice [52,53]
	NI	+	+	-	-	Rhs-family protein	NI
	NI	+	+	-	-	Rhs-family protein	NI
	NI	+	+	-	-	Phosphotriesterase	NI
	NI	+	+	-	-	Hypothetical protein	NI
	NI	+	+	-	-	Hypothetical protein	NI
	orf7 (*Photorhabdus* )	+	+	-	-	SinR-like, HTH transcription factor	NI [54]
	NI	+	+	-	-	Putative cytoplasmic protein	NI
	Pseudo	+	+	-	-	NI	NI
	Pseudo	+	+	-	-	NI	NI
	*tcfA*	+	+	-	-	Fimbrial protein	Increased expression with increased salinity, non virulence in INT-407 cells [55]
	*tcfB*	+	+	-	-	Fimbrial protein	Increased IgG-tcfB in patients with *S.* Typhi [56]
	*tsaC*	+	+	-	-	Fimbrial usher protein	No effect on adhesion to mice monolayers [57]
	*tcfD*	+	+	-	-	Fimbrial protein	NI [46]
	*rnhA-dnaQ* like	-	+	-	-	DNA polymerase 3 epsilon subunit ribonuclease H	NI [58]
	NI	-	+	-	-	Ribonuclease HI	NI
	*gloB* like	-	+	-	-	Hydroxyacylglutathione hydrolase	methylglyoxal degradation [46]
	*mltD*	-	+	-	-	Membrane-bound lytic murein transglycosylase D	Enchance virulence in *Vibria anguillarum* in zebrafish [59]
	NI	-	+	-	-	Methyltransferase UbiE/COQ5	Ubiquinone/ menaquinone biosynthesis [60]
	*yafD*	-	+	-	-	AP like endonuclease	Egg albumen resistance [7]
	NI	-	+	-	-	Putative drug efflux protein	NI
	NI	-	+	-	-	Hypothetical oxidoreductase	NI
	*dkgB*	-	+	-	-	2, 5 didehydrogluconate reductase B	Detoxing response to hyperosmotic solution [61]

SPI-6 distinguishes between *S*. Derby and *S*. Mbandaka strains. This island has also been extensively studied for a role in pathogenicity, and displays the largest amount of diversity in gene complement between *S*. Derby and *S*. Mbandaka. Interestingly the majority of the diversity in SPI gene complement between *S*. Derby and *S*. Mbandaka is additional genes in *S*. Derby isolates. NI signifies a lack of information on the ORF.

### Variation in SPI-3 between other serovars and *S.* Derby and *S.* Mbandaka

SPI-3 is highly variable, between *S.* Typhimurium 14028 and *S.* Choleraesuis SC-B67 the only region of homology is the insertion sequence tRNA-selC. SPI-3 from *S.* Derby D1 and D2 is an amalgamation of 19 SPI-3 genes from *S.* Typhimurium 14028, *S.* Dublin, *S.* Choleroaeasuis SC-B67 and *S.* Typhi CT18. *S.* Mbandaka M1 and M2 also contain a unique SPI-3 gene complement, containing 12 genes found in *S.* Typhimurium 14028, *S.* Choleraesuis SC-B67 and *S.* Typhi CT18. Unlike *S.* Derby D1 and D2, *S.* Mbandaka M1 and M2 have no SPI-3 genes in common with *S.* Dublin. STY4039 previously unique to *S.* Typhi CT18 is present in *S.* Mbandaka M1 and M2 and absent from *S.* Derby D1 and D2 (Table 1). The main region of variation between *S.* Derby D1 and D2 and *S.* Mbandaka M1 and M2 SPI-3 is at the start of the island where the complete *S.* Dublin SPI3 is present, this was shown previously for *S.* Derby 9813031, 0010160 and 0010158 [38,39]. This region contains seven genes relating to the adhesion structures, pili and fimbriae. *S*. Mbandaka M1 and M2 contains *rhuM* found in the SPI-3 of *S*. Typhimurium 14028; this sequence is absent from the SPI-3 of *S.* Derby D1 and D2. *S.* Derby D1 and D2 and *S.* Mbandaka M1 and M2 share 10 SPI-3 genes in common; this complement of genes is unique to these two serovars. Both serovars contain five virulence genes present in the SPI-3 of *S.* Typhimurium 14028 and *S.* Choleraesuis SC-B67. Both serovars lack the virulence gene *mgtC* which is present in *S.* Typhimurium 14028, *S.* Choloraesuis SC-B67 and *S.* Typhi CT18. In *S.* Typhimurium LT2 and 14028 *mgtC* was shown to be essential for intra-macrophage survival [62].

### Variation in SPI-5 between other serovars and *S.* Derby and *S.* Mbandaka

It has previously been shown that SPI-5 from *S.* Derby 9813031, 0010160, 0010158 and *S.* Ohio 9815932, 9714920, 9714922 contain an additional unnamed ORF, this ORF was present in both *S.* Derby D1 and D2 and *S.* Mbandaka M1 and M2 [37].

### Variation in SPI-6 between *S.* Derby and *S.* Mbandaka

SPI-6 is found in *S.* Typhi CT18, is 57 Kb in length and contains 59 genes. Between *S.* Derby D1 and D2 and *S.* Mbandaka M1 and M2, 24 genes that were not found in other islands on PAI-DB were identified by Glimmer3 (Table 1) [6]. The annotations here were taken from NCBI BLASTn results, many of which were hypothetical or putative in description. SPI-6 also shows the largest variation between *S*. Derby D1 and D2 and *S.* Mbandaka M1 and M2 outside of prophage and SPI-23 nucleotide sequences. SPI-6 in D2 had 8 unique genes at the C terminus of the positive strand that were not found in the other isolates. This contains an AP like endonuclease gene related to egg albumin resistance, *yafD*[63]. *S*. Derby has been isolated from inside of eggs while *S*. Mbandaka has been shown to grow slower than other *S. enterica* serovars in albumin [7]. The remainder of the island showed no variation amongst isolates of the same serovar. Seven *S*. Typhi CT18 genes were absent from both serovars, these were STY0300-STY303, STY0342, STY0350 and STY03351. *S*. Derby D1 and D2 SPI-6 contained 8 genes from *S.* Typhi CT18 that were absent from *S.* Mbandaka M1 and M2. *S.* Mbandaka M1 and M2 SPI-6 contained 20 genes from *S.* Typhi CT18 that were absent from *S.* Derby D1 and D2.

The variation in the gene complement of SPI-6 in *S*. Derby and *S*. Mbandaka is of particular interest with regards to host adaptation. *S.* Derby possess the gene *sirA* which corresponds with ORF STY0300 in *S*. Typhi CT18, that codes for a transcription factor linked with regulation of the TTSS encoding SPI-1 when inside a mammalian host [8,9]. Interestingly mutants for *sirA* in *S*. Typhimurium LT2 were attenuated in a bovine gastro-enteritis model, but were still proficient at causing typhoid fever in a mouse model [47,48,50,64]. The SPI-6 of *S*. Mbandaka also contains a gene *sciN* which is an outer membrane lipoporotein essential for biofilm formation in *E. coli* which is absent from *S*. Derby [64].

### Variation in SPI-9 from *S.* Typhi CT18

The alignment between SPI-9 of *S.* Derby D1 and D2 and *S.* Mbandaka M1 and M2 showed 100% sequence homology. SPI-9 from *S.* Typhi CT18 contains four genes as do the islands in *S.* Derby D1 and D2 and *S.* Mbandaka M1 and M2, though there is a difference in ORF length. STY2875 is at the start of the island and is 10.8 kb in length, in both *S.* Derby D1 and D2 and *S.* Mbandaka M1 and M2 an additional region of 595 bp is found between bases 3056 and 3057. The other three ORFs are truncated at the beginning of each sequence by 162 bp.

### Variation in SPI-11 from *S.* Derby and *S.* Mbandaka

The same eight genes from SPI-11 of *S.* Choleroeaesuis SC-B67 are absent in *S.* Derby D1 and D2 and *S.* Mbandaka M1 and M2. One of these genes is the effector protein *sopB*, which has been implicated in fluid secretion in calf ileal loops and is essential for enteropathogenicity of *S*. Dublin [65,66] although, as previously mentioned, a homolog to this gene was found elsewhere on the chromosome. SPI-11 also encodes the gene *pagC,* an envelope protein which increases survival within mouse macrophage [67].

### Variation in SPI-12 between *S.* Derby and *S.* Mbandaka

SPI-12 is an 11 Kb island first identified in *S.* Choleraesuis SC-B67. The island is inserted at a tRNA-Pro. The insertion sequence was present in both *S.* Derby D1 and D2 and *S.* Mbandaka M1 and M2, though no genes were adjacent to this site. Alignment of the whole SPI-12 island from *S.* Choleroeaesuis SC-B67 with D1, D2, M1 and M2 identified homologs for all the genes in each sequence, not in a single unit, but spread across the chromosome.

### Variation in CS54 between *S.* Derby and *S.* Mbandaka

CS54 identified from *S.* Typhimurium 14028 is associated with virulence and shows variation between isolates of the same serovar. All isolates lack the virulence genes *shdA* and *ratB*, and the untested gene *ratC*. M2 lacks the whole island with only the insertion sequences present (Table 1). This region in D1, D2 and M1 contains three genes *ratA*, *sivI* and *sivH* previously identified in CS54 of *S.* Typhimurium 14028. CS54 was previously described in *S.* Derby strain De1, in this instance *ratB* was also found, a gene essential for the colonisation of the cecum in BALB/c mice by *S.* Typhimurium IR715 [68].

### A novel *Salmonella* pathogenicity island designated SPI-23

**Figure 2:**
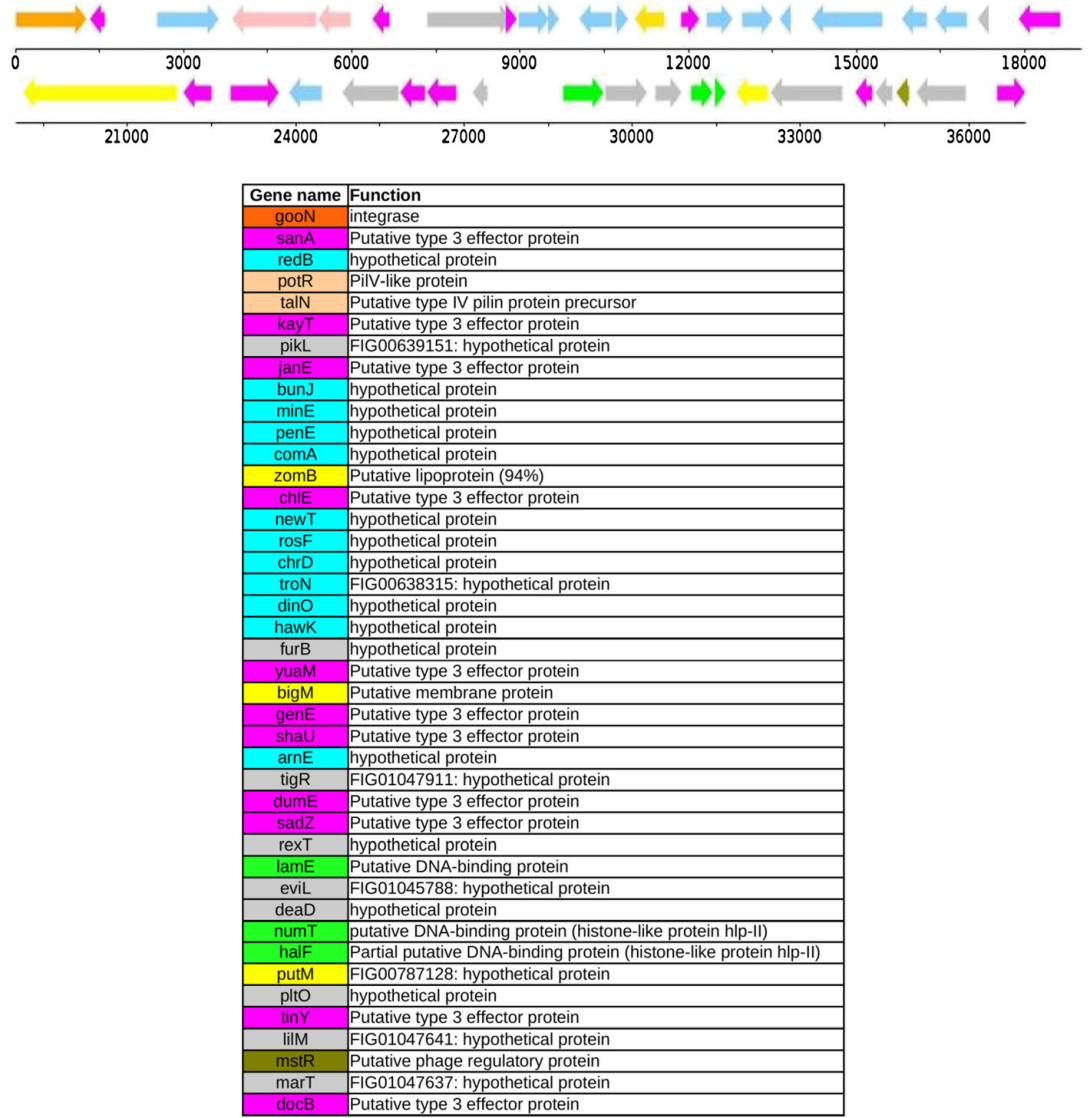
**SPI-23 from *S. *Derby D1 and D2. **SPI-23 is a putative pathogenicity island, in *S*. Derby it is 37 Kb long and contains 42 ORFS. Gene colours reflect putative function; orange, identifies a phage protein, blue a novel hypothetical protein, light pink identifies pili associated proteins, green a DNA binding protein, yellow a membrane protein, brown a regulatory protein and grey a conserved hypothetical protein. Dark pink identifies an ORF that was predicted to be an effector protein by SIEVE with a p-value of 0.05 or lower.

A new genomic island with a putative role in pathogenesis, SPI-23, was discovered in this study on the chromosome of D1 and D2 between bases 2027348–2065972 and 2052685–2089962 respectively flanked by tRNA-asn (GTT) and a hypothetical protein, *docB* (Figure 2). SPI-23 is composed of 42 ORFS with an overall GC composition of 38% differing largely from the 51% of the *S.* Derby genome. SPI-23 is completely missing from *S.* Mbandaka. Of the 42 ORFs 28 were of hypothetical status, of which, 17 contained no homology with an entry in the NCBI nucleotide database (accessed on 1/10/12). The island contains two genes, *potR* and *talN*, both implicated in type IV secretion and the production of pili. There is a single gene, *zomB*, predicted here to encode a lipoprotein. We also find five DNA binding proteins, *furB*, *lamE*, *halF*, *mstR* and *numT* and two putative membrane protein *bigM* and *putM*. SPI-23 contains a single NUDIX hydrolase, a very ubiquitous protein family involved in a multitude of regulator processes [10].

SIEVE effector protein predictor identified ten ORFs (*sanA, janE, chlE, yuaM, genE, shaU, dumE, sadZ, tinY* and *docB*) in SPI-23 of *S*. Derby D1 and D2 with a p-value of 0.05 or lower corresponding to a Z-Score of 1.5 or higher (Table 2)[69]. *docB,* encoding a putative effector protein was identified by RAST as a putative endoprotease and was found here to be conserved in *S.* Derby D1 and D2, *S*. Mbandaka M1 and M2, *S*. Agona SL483, *S*. Dublin ct02021853, *S*. Gallinarum SGG1, *S*. Enteritidis P125109, *S.* Newport SL254, and *S*. Typhimurium LT2. The functional prediction of *docB* fits with the function of other type III secretion effector proteins which have a cysteine protease activity [70]. The high number of potential type III secretion system effector proteins makes SPI-23 a strong candidate for classification as a pathogenicity island. The acquisition of this sequence could be responsible for the modulation of the host’s cell, cytoskeleton, immune response and intracellular signalling [71,72]. Though it is not possible to determine here if SPI-23 plays a role in defining the host range of *S*. Derby, the high number of potential effector proteins has identified it as a very interesting region for future experimental study of host adaptation.

**Table 2:** **Comparison of the annotation results for SPI-23 from different *S. enterica *serovars**

**Gene name**	***S*. Derby function**	**Sieve z Score**	***S*. Agona function**	**Sieve z Score**	***S. *Dublin function**	**Sieve z Score**	***S. *Gallinarum function**	**Sieve z Score**
*gooN*	Phage intergrase	0.27	Phage intergrase	−0.05	Phage intergrase	0.27	Phage intergrase	0.27
*sanA*	Exported protein	1.56	Exported protein	1.56	Exported protein	1.40	Exported protein	1.56
*redB*	No Matches	1.43	-	-	-	-	-	-
*-*	-	-	hypothetical protein	0.84	-	-	-	-
*-*	-	-	-	-	threonine operon leader	1.09	threonine operon leader	1.09
*-*	-	-	-	-	hypothetical protein	1.21	hypothetical protein	0.73
*-*	-	-	hypothetical protein	1.31	-	-	-	-
*-*	-	-	-	-	hypothetical protein	1.68	hypothetical protein	2.06
*-*	-	-	-	-	hypothetical protein	2.44	hypothetical protein	2.44
*-*	-	-	RelA/SpoT	0.25	-	-	-	-
*-*	-	-	hypothetical protein	0.71	-	-	-	-
*potR*	prepilin-type N- cleavage/methylation domain protein	0.90	prepilin-type N- cleavage/methylation domain protein	0.72	-	-	-	-
*-*	-	-	-	-	Pil-v like	1.82	Pil-v like	1.82
*-*	-	-	-	-	Pil-v like	0.75	Pil-v like	0.75
*talN*	Putative type 4 pilin protein	1.36	Putative type 4 pilin protein	1.03	Putative type 4 pilin protein	1.36	Putative type 4 pilin protein	1.36
*kayT*	Conserved Hypothetical	1.44	Conserved Hypothetical	1.71	Conserved Hypothetical	2.40	Conserved Hypothetical	1.66
*pikL*	Hypothetical 91% homology	0.38	Hypothetical 91% homology	0.38	Hypothetical 91% homology	1.44	Hypothetical 91% homology	0.33
*janE*	No Matches	1.51	-	-	-	-	-	-
*-*	-	-	hypothetical protein	1.06	-	-	-	-
*-*	-	-	hypothetical protein	0.98	-	-	-	-
*-*	-	-	-	-	hypothetical protein	0.49	hypothetical protein	0.49
*bunJ*	No Matches	1.35	-	-	-	-	-	-
*minE*	No Matches	0.13	-	-	-	-	-	-
*penE*	No Matches	1.25	-	-	-	-	-	-
*comA*	No Matches	1.40	-	-	-	-	-	-
*zomB*	Putative lipoprotein (94%)	−0.24	Putative lipoprotein (94%)	−0.05	Putative lipoprotein (94%)	1.22	Putative lipoprotein (94%)	1.22
*-*	-	-	hypothetical protein	0.69	hypothetical protein	0.89	hypothetical protein	2.61
*-*	-	-	hypothetical protein	1.22	hypothetical protein	0.30	hypothetical protein	−0.34
*-*	-	-	hypothetical protein	1.17	-	-	-	-
*-*	-	-	hypothetical protein	1.46	-	-	-	-
*-*	-	-	hypothetical protein	0.52	-	-	-	-
*-*	-	-	hypothetical protein	0.98	-	-	-	-
*-*	-	-	hypothetical protein	0.98	-	-	-	-
*-*	-	-	hypothetical protein	1.13	-	-	-	-
*-*	-	-	-	-	hypothetical protein	0.36	hypothetical protein	1.30
*-*	-	-	-	-	hypothetical protein	0.70	hypothetical protein	0.70
*-*	-	-	-	-	hypothetical protein	0.16	hypothetical protein	0.16
*chlE*	No Matches	2.02	-	-	-	-	-	-
*newT*	No Matches	0.82	-	-	-	-	-	-
*rosF*	No Matches	0.75	-	-	-	-	-	-
*chrD*	No Matches	0.15	-	-	-	-	-	-
*troN*	No Matches	0.50	-	-	-	-	-	-
*dinO*	No Matches	0.72	-	-	-	-	-	-
*hawK*	No Matches	1.26	-	-	-	-	-	-
*furB*	Hypothetical	0.39	-	-	-	-	-	-
*yuaM*	No Matches	1.67	-	-	-	-	-	-
*bigM*	Putative membrane protein (89%)	1.44	-	-	-	-	-	-
*genE*	No Function	2.06	-	-	-	-	-	-
*shaU*	No Matches	1.66	-	-	-	-	-	-
*arnE*	No Matches	0.82	-	-	-	-	-	-
*tigR*	Conserved Hypothetical	1.29	-	-	-	-	-	-
*dumE*	No Matches	1.92	-	-	-	-	-	-
*sadZ*	Hypothetical 88%	1.58	-	-	-	-	-	-
*rexT*	Pentatricopetide 90%	0.85	-	-	-	-	-	-
*lamE*	Putative DNA-binding protein	0.27	Putative DNA-binding protein	1.40	Putative DNA-binding protein	1.48	Putative DNA-binding protein	1.48
*eviL*	Conserved Hypothetical	0.68	Conserved Hypothetical	0.27	Conserved Hypothetical	1.49	Conserved Hypothetical	0.27
*deaD*	Conserved Hypothetical	1.33	Conserved Hypothetical	0.68	Conserved Hypothetical	0.85	Conserved Hypothetical	0.85
*numT*	putative DNA-binding protein (histone-like protein hlp-II)	1.31	putative DNA-binding protein (histone-like protein hlp-II)	1.43	putative DNA-binding protein (histone-like protein hlp-II)	0.25	putative DNA-binding protein (histone-like protein hlp-II)	0.25
*-*	-	-	-	-	DNA-binding protein H-NS	0.57	DNA-binding protein H-NS	0.68
*-*	-	-	-	-	hypothetical protein	1.33	hypothetical protein	1.33
*-*	-	-	-	-	hypothetical protein	1.31	hypothetical protein	1.31
*-*	-	-	-	-	hypothetical protein	0.98	hypothetical protein	0.98
*-*	-	-	-	-	hypothetical protein	1.18	hypothetical protein	1.07
*-*	-	-	-	-	hypothetical protein	0.93	-	-
*-*	-	-	-	-	hypothetical protein	0.73	hypothetical protein	0.73
*halF*	Partial putative DNA-binding protein (histone-like protein hlp-II)	0.98	-	-	-	-	-	-
*putM*	Putative membrane protein	0.93	-	-	-	-	-	-
*pltO*	Conserved Hypothetical	0.73	-	-	-	-	-	-
*tinY*	Conserved Hypothetical	2.23	-	-	-	-	-	-
*lilM*	Hypothetical 90%	1.41	-	-	-	-	-	-
*mstR*	Putative phage regulatory protein	0.74	Putative phage regulatory protein	1.90	Putative phage regulatory protein	1.71	Putative phage regulatory protein	1.71
*marT*	Hypothetical 99%	−0.14	-	-	-	-	-	-
*-*	-	-	hypothetical protein	0.71	-	-	-	-
*-*	-	-	hypothetical protein	0.74	-	-	-	-
*docB*	Putative endoprotease 99%	1.81	Putative endoprotease 99%	1.81	Putative endoprotease 99%	1.81	Putative endoprotease 99%	1.81
*-*	-	-	hypothetical protein	−0.14	-	-	-	-
*-*	-	-	hypothetical protein	1.11	-	-	-	-
*-*	-	-	TPR domain protein, putative component of TonB system	0.56	-	-	-	-
*-*	-	-	hypothetical protein	0.49	-	-	-	-
*-*	-	-	hypothetical protein	0.75	-	-	-	-
*-*	-	-	putative P4-type integrase	1.18	-	-	-	-
	-	-	hypothetical protein	1.30	-	-	-	-
*-*	-	-	hypothetical protein	0.93	-	-	-	-
*-*	-	-	hypothetical protein	−0.05	-	-	-	-
*-*	-	-	hypothetical protein	0.53	-	-	-	-

This Table shows the comparative structure and gene content of SPI-23 in the chromosome of different serovars. SIEVE Z-scores above 1.5 indicate a potential type III effector protein. Functions are taken from RAST, or where no function was given, the highest hit on NCBI BLASTn. Provisional gene names are given for ORFs in SPI-23 of *S*. Derby; this does not conflict with existing gene names, which have been used where possible.

### Comparison of SPI-23 from *S.* Agona SL483, *S.* Dublin ct02021853 and *S.* Gallinarum SGG1 with SPI-23 found in *S.* Derby D1 and D2

**Figure 3:**
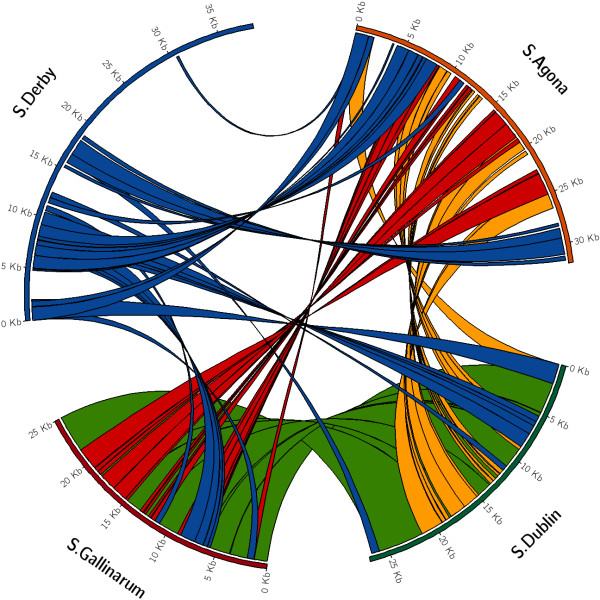
**SPI-23 four way nucleotide comparison. **Four way comparison of the nucleotide sequence of SPI-23 from *S*. Derby D1*, S*. Agona SL483, *S*. Dublin CT02021853 and *S*. Gallinarum RKS5078. *S*. Derby D1 possess the largest SPI-23 (37 Kb) island of the sequenced strains available on NCBI genome and the most novel in nucleotide sequence. Over 60% of the nucleotide sequence of SPI-23 in *S*. Derby D1 is unique and contains no entry on NCBI nucleotide database.

There were no genes between *gooN* and *docB* in *S.* Enteritidis P125109 even though NCBI BLASTn showed 100% sequence homology with 17 genes from SPI-23 of *S.* Derby D1 and D2. A four way comparison between SPI-23 excised from the genomes of *S.* Agona SL483, *S.* Dublin CT02021853, *S.* Gallinarum SGG1 and *S*. Derby D1 was performed (Figure 3). The differences in SPI-23 between *S.* Agona SL483 and *S.* Derby D1 and D2 are dispersed across the island in four sections (Table 2). *S.* Agona SL483 contains seventeen unique genes and lacks twenty two genes when compared to the SPI-23 of *S.* Derby D1 and D2. All of the genes unique to *S.* Agona SL483 with the exception of three are of hypothetical status, *relA* a GDP/GTP pyrophosphokinase, a putative component of the TonB system and a P4 type intergrase. SPI-23 in *S*. Agona SL483 contains only four genes that are likely candidates for type III secretion system effector proteins (*sanA, kayT, mstR* and *docB*). All four genes are identical in nucleotide sequence to that of *S*. Derby D1. Serovars *S.* Dublin CT02021853 and *S.* Gallinarum SGG1 have identical sequences for SPI-23. There are fifteen genes in the SPI-23 of these two serovars that are not found in either *S.* Derby D1 and D2 or *S.* Agona SL483 and fifteen which are found in all five serovars. Only two of the hypothetical genes found in *S.* Agona SL483 and not *S.* Derby D1 and D2 are found in the SPI-23 of *S.* Dublin CT02021853 and *S.* Gallinarum SGG1. The SPI-23 of *S.* Dublin CT02021853 and *S.* Gallinarum SGG1 contains four unique genes that are not of hypothetical status. This comprises two *pilV*-like proteins, a DNA –binding protein HNS and a threonine operon leader protein. Both *S.* Dublin CT02021853 and *S.* Gallinarum SGG1 contain eight putative type III secretion system effector proteins, three of these are unique genes to these two sequences and are absent from *S.* Derby D1, D2 and *S*. Agona SL483. Two putative effector proteins are different between the SPI-23 sequences of *S.* Dublin CT02021853 and *S.* Gallinarum SGG1, *sanA* is present in *S.* Dublin CT02021853 but not identified as a putative effector protein and similarly a hypothetical gene in *S*. Dublin CT02021853 and *S.* Gallinarum SGG1. Interestingly SIEVE predicts the gene *kayT* as a type III secretion system effector protein from the amino acid sequences of *S*. Agona SL483, *S.* Dublin CT02021853 and *S*. Gallinarum SGG1 but not that of *S*. Derby D1 or D2. Similarly *sanA* is identified as a candidate effector protein in all sequences with the exception of *S.* Dublin CT02021853.

### Prophage

Bacteriophages are viruses that infect bacteria, integrating into the bacterial genome in order to replicate; in this form they are known as prophage. As a result of phage insertion the genome gains a substantial amount of foreign sequence, much of which encodes phage structural proteins. However, some phage carry cargo genes which convey a pathological advantage to the recipient [71]. The process of lysogenic conversion prevents the prophage from destroying the host through maturation of progeny. The cargo genes and prophage remnants are therefore retained within the bacterial lineage, undergoing genetic mutation, drift and selection [73].

PHAST identified distinct complements of intact prophage and remnant prophage regions between *S.* Derby and *S.* Mbandaka [74]. All isolates contain four phage regions, sharing only the remnants of a BcepMu phage in common. This remnant is identical in all strains, suggesting that the integration and degradation of this phage predates the split between *S.* Derby and *S*. Mbandaka. *S*. Mbandaka isolates contain the same prophage regions in the same locations along the chromosome. These comprise one intact prophage, resembling phage P2, two questionable prophage, similar to L413c and Epsilon34 and one incomplete prophage BcepMu. *S*. Derby isolates differed on the location of the prophage within the chromosome and the number of genes in all four phage regions. No ambiguous bases were identified in these regions. The partial prophage resembling SFV contains one additional ORF in D1 than in D2 and occupies the same region that the complete prophage of SFV occupies in D2. Whereas the complete copy of SFV in D1 occupies the position of the complete prophage in D2 and contains one fewer ORF. The BcepMu partial in D1 contains two additional ORFs than that found in D2. In D1 the intact prophage resembling ST64B comprises three additional ORFs than that found in D2, they occupy the same chromosomal region. ST64B is of particular interest as its homolog in *S.* Typhimurium SL1344 contains a gene with homology to a type III secreted effector protein Sske2, mutants of which have shown to have reduced pathogenicity in a bovine model [75]. *S*. Derby contains an intact version of IN0, a transposon identified from *Pseudomonas aeruginosa*.

### *S.* Derby and *S.* Mbandaka contain unique CRISPR spacer sequences

CRISPR operons convey an adaptive immunity against plasmids and bacteriophage to a broad range of archaeal and bacterial species. This is achieved through integration of unique regions of foreign DNA into the prokaryotic chromosome. Subsequent expressions of these fragments interfere with foreign nucleic acid, through complementation [76,77]. The spacer sequences within a CRISPR operon reflect the historical interaction between the lineage of a strain and foreign DNA elements. The efficacy of invasion and ecological distribution of bacteriophage, transposons and plasmids have been found to associate with particular hosts and environments [78,79]. Hence the different genomic complement of prophage and CRISPR operon elements in *S.* Derby and *S.* Mbandaka could reflect their particular niche or even define their niche within a specific group of livestock species.

*S.* Derby D1 and D2 contain four CRISPR operons each, with 34 and 35 spacers respectively. *S.* Mbandaka M1 contained two CRISPR operons with 25 spacers. M2 contains three CRISPR operons with 27 spacers. With the exception of two spacers, the sequences are completely unique to each serovar. *S.* Derby isolates contain four CRISPR spacer operons, the smallest contains only one sequence with the largest containing 25 spacers. D2 contains two additional spacer sequences and half of a much larger spacer than D1. *S.* Mbandaka isolates differ on the number of spacers they each contain; M1 contains two operons while M2 contains three. The majority of spacers are homologous between the isolates, with M2 containing four additional spacers. M2 CRISPR operon 2 and 3 contain all of the spacer sequences in M1 CRISPR operon 1. All spacer sequences can be found in the supplementary materials [Additional file 1].

We have already shown that *S.* Derby strains contain seven functionally unique CRISPR operon proteins. The lack of functional homolog in *S*. Mbandaka, leaves it without a functioning CRISPR operon. CRISPRdb shows here that *S.* Mbandaka strains contain arrays of CRISPR spacer sequences; these may be remnant from when *S*. Mbandaka had a fully functioning CRISPR operon. *S*. Mbandaka may now be susceptible to those phage and plasmid for which it once had resistance; this could reflect the loss of positive selection pressure on the operon from the surrounding environment.

### Estimating the time since the divergence of *S*. Derby and *S*. Mbandaka

Whole genome alignment and SNP calling across CDS nucleotide sequences was used to estimate the years since divergence of D1, D2, M1 and M2. Interestingly the time since the divergence of *S*. Derby and *S*. Mbandaka is estimated at between 182,291 and 625,000 years, based on an average of the Ks values for all four pair-wise comparisons of *S*. Derby and *S*. Mbandaka isolates ranging between 0.015 and 0.019. The divergence of *S*. Derby and *S*. Mbandaka coincides with the estimated time of the divergence of all domesticated pig species, approximately 500,000 years ago [80-83]. The time since the split between D1 and D2 was estimated at between 350 and 1200 years ago based on 31 synonymous SNPs spread across 923506 synonymous positions. The isolates M1 and M2 are estimated to have diverged between 1271 to 4357 years ago based on 118 synonymous SNPs spread across 965114 synonymous positions.

## Conclusions

We estimate here that *S*. Derby D1 and D2 diverged from *S*. Mbandaka M1 and M2 between 182kya and 625kya, during this period these serovars appear to have adapted towards two distinct ranges of host species. Comparative functional genomics has alluded to several mechanisms that could contribute towards distinct host adaptations of *S*. Derby D1 and D2 and *S*. Mbandaka M1 and M2. Most noteworthy of these differences are the diversity in SPI-6 gene complement and the discovery in the chromosome sequence of *S.* Derby, of a new 37 Kb genomic island, SPI-23 encoding 42 ORFS, ten of which are putative TTSS effector proteins. The absence of functional homologs to several CRISPR operon genes in the chromosome sequences of *S*. Mbandaka may reduce the fitness of the serovar in environments laden with actively integrative foreign genetic elements. The increased gene dosage of the Csg-biofilm operon and the Ycd-swarming operon in *S*. Mbandaka could make the implementation of these two behaviours more readily achievable. Both of these behaviours are considered stress responses. *S*. Mbandaka also possesses an operon pertaining to the uptake and metabolism of D-galactonate into glycolysis which is absent from the chromosome of *S*. Derby.

The genetic background in which the function of the genes discussed here have been characterised is non-isogenic to the chromosome of *S*. Derby D1 and D2 and *S*. Mbandaka M1 and M2. Due to the different context these genes are found in, firm conclusions on the function of these genes in these specific serovars can only be formed through further biological experimentation.

## Methods

### Bacterial strains and culturing

The original isolates were stored at RT on Dorset egg slopes from which bead stocks were made with HIB + 30% glycerol, samples were frozen to −80°C in 2010 and remained frozen throughout the study. Unless stated otherwise, strains were grown for 16 hours aerobically either on LB agar plates or in liquid broth vigorously agitated at 220 rpm.

### DNA extraction, genome sequencing and assembly

DNA was extracted from 3 ml overnight cultures as per manufacturer’s instructions (Invitrogen EasyDNA kit). Sequencing was performed by the AHVLA Central Sequencing Unit, Weybridge. A Roche GSFLX titanium 454 pyrosequencer was used to produce rapid and paired-end libraries for whole genome DNA preparations of *S.* Derby D1 and D2 and *S*. Mbandaka M1 and M2. Roche protocols were used in all stages of sequencing. Paired-end library inserts were between 4 Kb to 9 Kb, containing 20,000 to 89,000 reads each. The rapid libraries contained between 69,000 and 173,000 reads each. Sequences were assembled *de novo* using Newbler v2.5. Scaffolds were reordered in ACT v9.0 in reference to a DoubleACT v2 comparison file of each genome with D1; D1 was chosen as the assembly consisted of a single scaffold [84,85]. The final sequences were then formatted so as to begin at the gene *thrL*, in line with other published *Salmonella enterica* genomes.

### Automated annotation, metabolic model construction and comparative genomics

Genomes were annotated using the RAST annotation system performed on 9/10/12, backfilling of gaps and automatic error fixing were enabled. Functional comparisons were implemented using the SEED genome viewer v2 [86,87]. An automated metabolic reconstruction was also produced from the complete genome sequence using the ModelSEED server v1.0 [16]. Differences in *S.* Derby and *S*. Mbandaka models were identified through gene overlays on top of KEGG maps [88]. Reciprocal BLASTing was implemented in SEED genome viewer for each ORF that differed between isolates to identify functional homologs. The genomes were also compared through sequence homology. The population of “hypothetical” and “putative” genes were aligned with a cut off of 90% bi-directional amino acid sequence homology.

### Mobile genetic elements

SPIs were identified from the genomes of *S.* Derby and *S*. Mbandaka through alignment of the insertion sequences with the newly acquired genomes. SPIs for the serovars *S*. Choleraesuis B67 (SPI-1, 2, 3, 4, 11, 12) and 1240 (SPI-11), *S.* Derby (SPI-5) isolate not specified, *S.* Gallinarum SGG1 (SPI-13) and SG8 (SPI-14)[89], *S*. Dublin isolate not specified (SPI-3), *S*. Typhi CT18 (SPI-3, 6, 7, 8, 9, 10) *S*. Typhimurium LT2 (SPI-1, 5, 18) and 14028 (CS54, SPI-3 ) were acquired from PAI-DB website [11], these were aligned using DoubleACT with the newly isolated islands. From previously published annotated genomes, SPI-22 from *S*. Bongori [14] and SPI-15, 16 and 17 from *S*. Typhi CT18 were excised from the genome [36]. SPI-19, 20 and 21 were excised from the genome of *S. enterica* subspecies *arizonae* (IIIa) serotype 62:z4,z23:- [62]. In most cases the SPI from *S*. Derby and *S*. Mbandaka could be completely annotated through alignment using DoubleACT with the existing SPI. Where gaps were present the BLAST facility was first used on PAI-DB, when no results were obtained, the RAST annotation and NCBI BLASTn were used to annotate the genes, and extensive literature research was used to assign a putative role in pathogenesis. Prophage were identified and categorised as intact, questionable and partial using PHAST [90]. CRISPR spacers were identified using CRISPRfinder [75,91]. CRISPRdb BLAST facility was used to see if the spacers found in the newly sequenced genome were found within other bacterial species [92]. Spacer sets of the newly sequenced strains were also cross compared to elicit the historical differences in exposure to phage that has occurred since their divergence. Hypothetical proteins found in SPI-1 and SPI-23 were tested for potential roles as TTSS effector through implementation of SIEVE-SVM based TTSS effector protein predictor [93]. A Z-score above 1.5 was taken to reflect a good indicator of a type III effector protein. SPI-23 was identified and extracted from the publicly available genomes for *S*. Agona SL483, *S*. Dublin CT02021853 and *S.* Gallinarum RKS5078. The sequences were annotated using RAST and SEIVE. Sequences were compared using DoubleACT and ACT.

### Estimation of years since *S*. Derby and *S*. Mbandaka diverged

For each genome (D1, D2, M1 and M2), nucleotide sequences of the CDS identified in the RAST annotation were converted into a single concatenated FASTA file using Artemis [70]. Sequences were aligned in Mauve genome aligner [94]. Aligned sequence blocks were reassembled from the Mauve alignment. A multiFASTA file was made for each combination of the four genomes. DNAsp was used to identify the synonymous and non-synonymous positions and SNPs [16,95]. The years since the isolates diverged was estimated as described by Foster et al. 2009 using the following formula [96]:

$$X=\frac{\mathrm{Ks}}{\left(2\;Z\;Y\right)}$$

Where X is the years since divergence, Ks is the proportion of synonymous SNPs to synonymous sites, Z is the mutation rate per generation and Y the number of generations per year. The mutation rate for *S*. Typhi has been estimated at 1.6 x 10^-10^ mutations per nucleotide per generation, calculated over 20,000 generations [97]. This is very close to the calculation for the rate of mutation in *E. coli* of 1.4 x 10^-10^ per nucleotide per generation estimated over 20,000 generations [98]. The rate of mutation has been shown to be highly variable and dependent on the mutation rate phenotype of the lineage [99]. Here we use both rates calculated between *S.* Typhi generations as the lower limit and *E. coli* generations as the upper limit, with the assumption that the true rate sits somewhere between these two. This is based on the assumption that the variation in the mutation rate correlates with the phylogenetic relationship between the strains [100,101]. The number of generations per year (Y) is taken from the estimate produced for a wild population of *E. coli* of between 100 and 300. The denominator is multiplied by two as it applies to the number of SNPs between two genomes [102]. To estimate the time of divergence between *S*. Derby and *S*. Mbandaka an average was taken of the four possible Ks values for each of the four pair wise comparisons.

### Visualisation of sequence architecture

Genomic maps were constructed using CIRCOS circular visualization of data tool v 0.56 [103]. A program for calculating GC skew in R v2.11.0 using the library SeqinR v3.0-6 was modified from R graphical manual example “fragment of *E. coli* chromosome” [104-106]. The GC skew was calculated under a 1 kb window at a 200 bp interval. The RAST annotation files were de-constructed into four tracks, forward and reverse coding DNA and RNA. The SPI-23 comparison maps were constructed from modified DoubleACT outputs for each combination of *S*. Derby D1 SPI-23 and the genomes of *S*. Agona SL483. *S.* Dublin CT02021853 and *S.* Gallinarum RKS5078, with a 100 bp cut-off for width between non-homologus sequences.

## Competing interests

The authors declare that they have no competing interests.

## Authors’ contributions

MRH, VAAJ and MJW conceived the study and wrote the manuscript. DNA extractions, bioinformatics and analysis were performed by MRH. All authors read and approved the final draft.

## Supplementary Material

Additional file 1 Differences in gene and phage complement, the genes flanking the inversion, and the CRISPR spacer sequences, between isolates D1, D2, M1 and M2. Click here for file

## Data Availability

Genome annotations and sequences available under guest log in on the RAST website, accessible from: http://rast.nmpdr.org/ using the following RAST IDs: D1 [RAST: 28144.16], D2 [RAST: 28144.17], M1 [RAST: 192954.16] and M2 [RAST: 192954.17].
